# Intracranial extension of an intramuscular haemangioma of superior rectus: case report and literature review

**DOI:** 10.1186/s12886-022-02429-4

**Published:** 2022-05-23

**Authors:** Saud Al-Johani, Arwa Al-Romaih

**Affiliations:** 1 Department of Ophthalmology, College of Medicine, Imam Abdulrahman Bin Faisal University, Dammam, Saudi Arabia; 2 Department of Ophthalmology, College of Medicine, King Saud University, P.O. Box 245, Riyadh, 11411 Saudi Arabia

**Keywords:** Intramuscular haemangioma, Extraocular muscle, Intracranial extension

## Abstract

**Background:**

Intramuscular haemangiomas are rare, benign vascular tumours that represent < 1% of all haemangiomas. When involving the extraocular muscles, haemangiomas are extremely rare, with only nine cases reported in the literature; to date there are no reported cases of extraocular muscle haemangiomas extending into the brain.

**Case presentation:**

A 6-year-old boy with a two-week history of a painless swelling and erythema on the upper eyelid. On examination, the patient had restricted extraocular motility in all directions of gaze. In addition, the eye appeared proptotic, with chemosis and hyperaemia of the conjunctiva. Visual acuity and intraocular pressure were normal. Orbital computed tomography imaging showed a mass in the left superior rectus, with heterogeneous enhancement following contrast administration. Incisional biopsy revealed an intramuscular haemangioma of the superior rectus muscle with capillary-type vessels. The patient received an intralesional steroid, which improved the condition for a few months; however, the lesion later recurred and included an intracranial extension.

**Conclusion:**

This case represents the first reported case of intracranial extension of intramuscular haemangioma of extraocular muscle.

## Background

Intramuscular haemangiomas (IMHs) are a rare type of tumour, accounting for less than 1% of the total number of haemangiomas; their occurrence in extraocular muscle is extremely rare [1–3]. IMHs are considered to be benign, congenital, slow growing neoplasms that can remain undetected for a long time, only becoming apparent when sudden growth causes symptoms. The most common initial presentation is a palpable mass. The tumours are benign, and in contrast to the cutaneous haemangiomas of infancy, they never spontaneously regress. There are just two management options: managing it with systemic steroids or surgical excision [2]. However, even after wide resection of a cuff of normal muscle around the tumour, the rate of recurrences ranges from 9 to 28% [4]. This is unique case study describes the unusual presentation of an intramuscular haemangioma of the superior rectus muscle with intracranial extension in a child. A review of the literature is also included.

## Case presentation

**Fig. 1:**
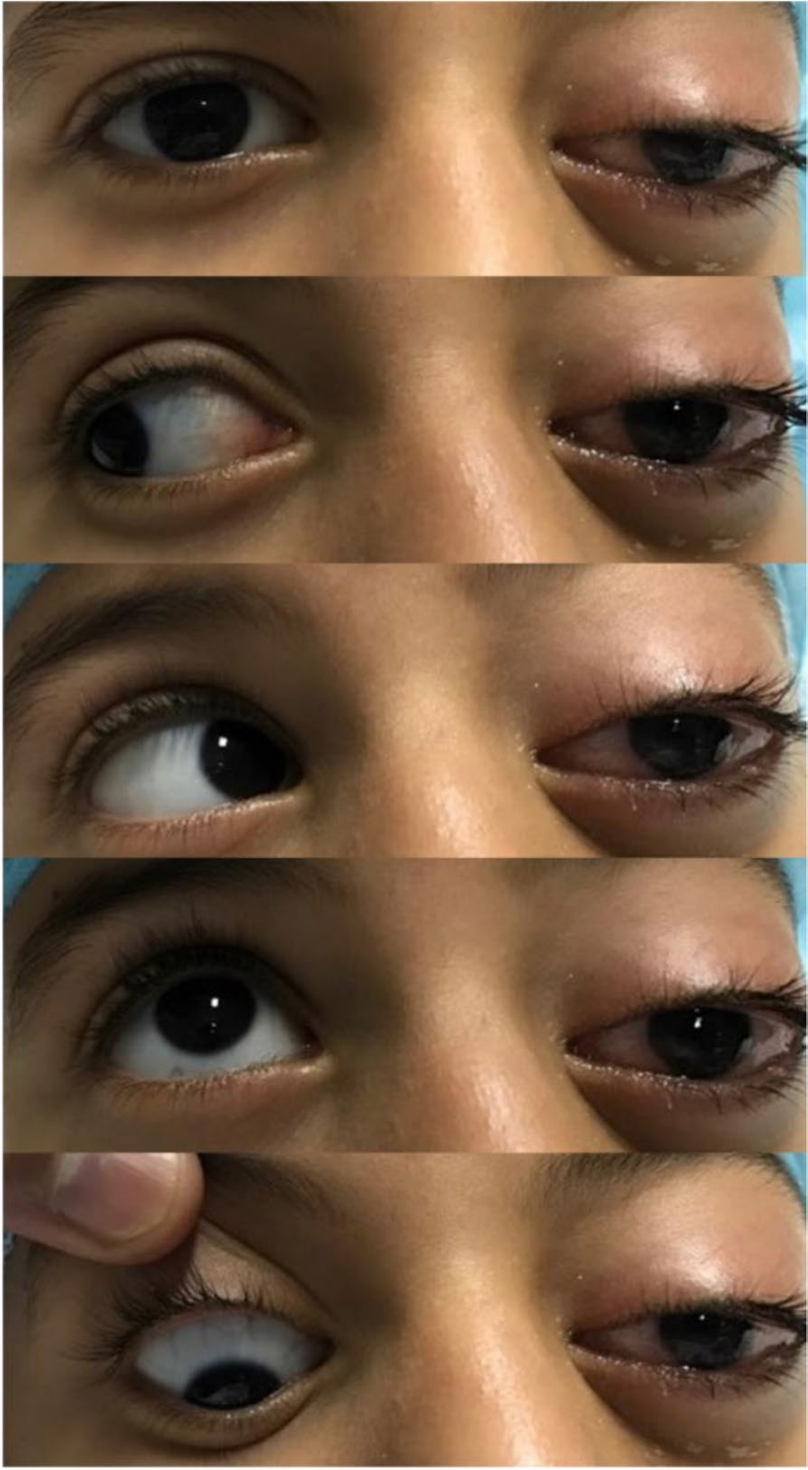
Clinical appearance of the haemangioma in the left eye, proptosis and EOM movement restriction

A 6-year-old boy, not known to have any medical illness, presented to our hospital complaining of painless redness and swelling of the left upper eyelid with proptosis that had developed over two weeks. Upon examination, best corrected visual acuity (BCVA) of the right and left eyes were 20/20 and 20/30 respectively. Intraocular pressure (IOP) of both eyes was 12 mmHg, and the left eye showed mild upper lid swelling, proptosis with restricted extraocular (EOM) movement in all gaze directions. Examination of the anterior segment shows mild chemosis with hyperaemia, clear cornea and normal fundus (Fig. 1).

**Fig. 2:**
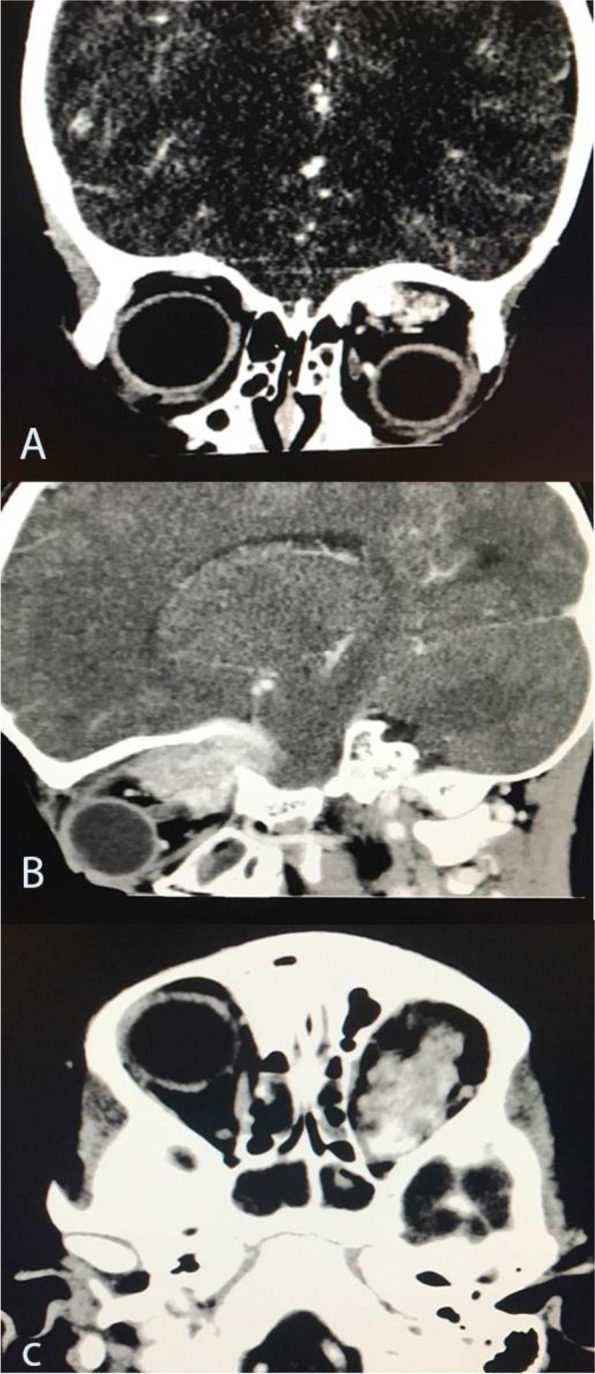
Orbital CT scan with coronal (**A**), sagittal (**B**) and, axial views (**C**) showing well demarcated mass along the left superior rectus muscle

A computed tomography (CT) scan with contrast revealed a left orbital soft tissue mass measuring 3.7 × 1.7 cm. The isodense mass in the muscle displaced the superior rectus and left globe downwards and anteriorly. The mass showed heterogeneous enhancement. There was bone erosion to the superior left orbital bone ridge (roof) extending to the brain (Fig. 2).

**Fig. 3:**
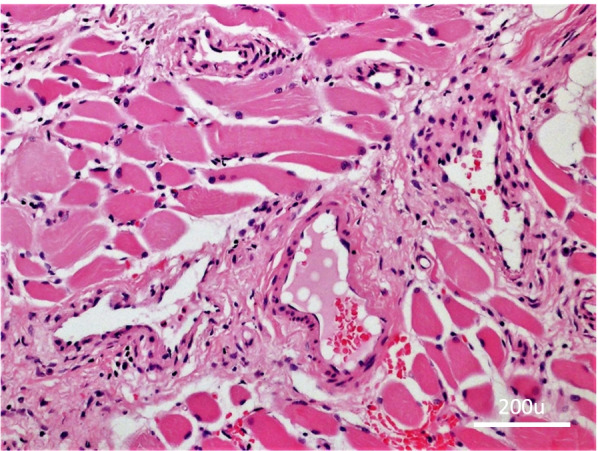
Histopathologic analysis of the biopsy, shows predominantly small capillary-like vessels mixed with muscular fibres (hematoxylin–eosin stain; original magnification, × 200)

Under general anaesthesia, a biopsy was performed through the infrabrow region using a subperiosteal approach to the midsuperior orbit. The histopathologic analysis revealed large, variably sized vessels infiltrating between muscular fibres (Fig. 3).

A diagnosis of a capillary-type intramuscular haemangioma of the superior rectus muscle was made. The patient received one dose of intralesional triamcinolone acetate (TA) (40 mg /ml). The lesion regressed, proptosis resolved, and full extraocular movement was restored, with normal BCVA and IOP.

**Fig. 4:**
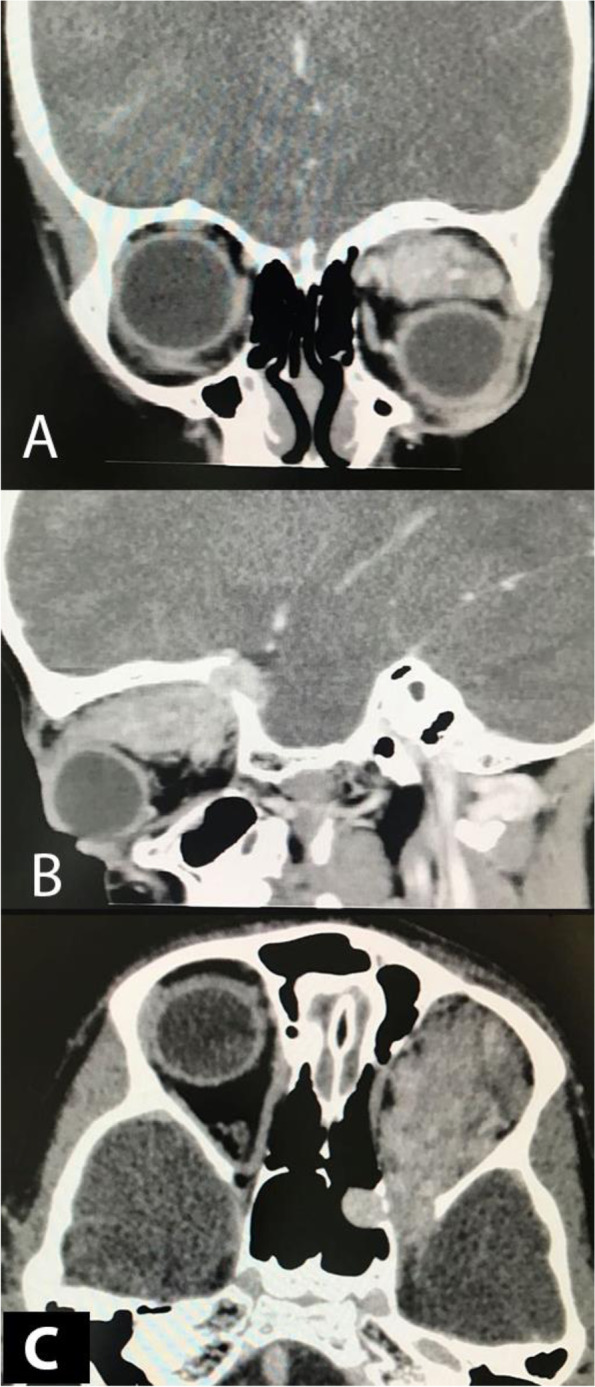
Orbital CT scan with coronal (**A**), sagittal (**B**) and axial views (**C**), showing the recurrence of the intramuscular haemangioma with intracranial extension

Few months later, the patient came back complaining of the same symptoms in the same eye. Upon examination of the left eye the BCVA was 20/40, the IOP was 14 mmHg, and there was complete restriction of EOM. Another dose of TA was given, but there was no improvement. A CT scan of the orbit showed the tumour had recurred; it was accompanied by bone destruction and an intracranial extension that involved the sphenoid sinus. Furthermore, following the administration of contrast, CT revealed strong enhancement of the tumour (Fig. 4). The patient was referred to neurosurgery for a complete surgical excision of the tumour. Unfortunately, the patient was lost to follow up after referring to neurosurgery department.

## Discussion

IMHs constitute less than 1% of all haemangiomas and are usually located in the skeletal muscles of the trunk or limbs; they rarely occur in the head and neck region [5]. Isolated IMHs of a single extraocular muscle are exceedingly rare [3]. The histological characteristics of IMH have been sub-classified by Allen and Enzinger to be capillary type (small vessel), cavernous type (large vessel) and mixed types, with frequencies of 50, 29, and 21%, respectively [1]. The exact aetiology is unknown, although congenital, traumatic and hormonal theories have been proposed [2].

Only nine such cases of IMHs involving extraocular muscles are reported in the literature (Table 1). The range of ages affected by IMH is wide and there is no apparent relationship between prevalence and gender. Reported symptoms include a painless, localised slowly enlarging mass, gradually progressive proptosis, eyelid swelling and diplopia. None of the nine reported cases had included intracranial extension.

**Table 1:** Review of the literature of “intramuscular haemangiomas” in extraocular muscles

Year	Author	Patient’s age	Extraocular muscle involved	Clinical features	Histopathologic type	Treatment
2002	Christensen	21 yrs	MR, LR, IR, SO	Painless, slowly progressive, non-compressible retrobulbar lesion, no hyperemia	Mixed	Enucleation
2003	Kiratli	3 yrs	LR	Painless, slowly progressive upper eyelid swelling, no hyperemia	Capillary	Systemic steroids
2003	Kiratli	40 yrs	MR	Painless slowly progressive proptosis, eyelid oedema, conjunctival chemosis and dilated episcleral vessels	Mixed	Systemic steroids
2006	Kim	63 yrs	SR	Painless slowly progressive proptosis, upper and lower eyelids oedema, hypertropia, no erythema or chemosis	Cavernous	Strabismus surgery and systemic steroids
2009	Lee	31 yrs	MR	Painless slowly progressive proptosis, optic nerve oedema	Cavernous	Excision with posterior decompression
2014	Charles	25 yrs	IO	Painless lower eyelid mobile, non-tender mass, hyperglobus	Capillary	Observation
2017	Mehta	11 yrs	MR	Painless slowly progressive irregular bluish mass on medial rectus muscle, limited extraocular movements on supraduction, infraduction and adduction	Cavernous	Excision
2019	Gade	61 yrs	MR	Painless slowly progressive proptosis, diplopia, afferent pupillary defect, decrease vision	Mixed	Excision with posterior decompression
2020	Tabuenca		IR		Cavernous	Fractionated stereotactic radiotherapy
2021	Bentham	26 yrs	MR	Painless slowly progressive proptosis, hypoglobus, limited upgaze and abduction, lower lid retraction		Observation
2021	Our case	6 yrs	SR	Upper lid swelling, proptosis with restricted extra ocular movement in all gazes	Capillary	Intra-lesional steroid

Christensen et al. [5] were the first to report IMH affecting the extraocular muscles. In that case, the tumour was large and compressing the optic nerve, which resulted in reduced visual acuity, for which enucleation of the eye was performed. Similar findings were reported by Lee et al. and Gade et al. Their patients underwent complete excision with posterior decompression of the tumour. The two patients recovered well, and did not experience any postoperative neurological deficits [4, 6]. Kiratli et al. reported two cases, a 3-year-old child and 40-year-old man, who both presented with proptosis and a swollen eyelid. Following a biopsy of the lesion, the man was treated with oral prednisone for three months, which resulted in a moderate clinical improvement, evidenced by the reduction of proptosis and eyelid oedema. A repeated magnetic resonance imaging (MRI) scan failed to show tumour regression [7]. Kim et al. describe the IMH presented in a 63-year-old man presented with diplopia; they report the involvement of the right superior rectus with large-angle hypertropia, and confirmed the IMH diagnosis by biopsy. A right superior rectus recession and inferior recuts resection was made, and systemic corticosteroids administered to improve the swelling [1]. Mehta et al. report on the case of an 11-year-old male child with a two-year history of a mass in the nasal aspect of the right eye and an associated decrease in EOM. The right medial rectus was involved. The mass was excised completely, and on histopathological examination, a purely cavernous type of IMH was noted [2]. One reported case was treated with fractionated stereotactic radiotherapy after the tumour recurred following post-surgical excision. The lesion responded well, exhibiting a reduction in size and symptoms [8].

The differential diagnosis of enlarged EOM includes thyroid eye disease, idiopathic inflammation, metastases and lymphomas. Other but less likely possibilities include amyloidosis, *Trichinella spiralis* infection, cysticercosis and dermoid cysts. Hence, to establish a definitive diagnosis of IMHs is difficult; it is only possible with biopsy and histopathology. Imaging features of IMHs often show a sharp demarcation, with bright areas within the tumours that indicate a combination of large vessels with stagnant blood and nonvascular tissue. Images also show areas of septated-striated high signal intensity and curvilinear areas of low signal intensity [1, 6, 7].

Many treatment modalities have been advocated for cases of cutaneous and extra-orbital skeletal haemangiomas; these include administering cryotherapy, radiotherapy, steroid or sclerosing agents and embolisation. However, at present, the optimal management approach remains total surgical excision that includes an adequate margin of surrounding healthy tissue [1, 5–7]. The drawback of implementing this approach in the extraocular region is it can result in an irreversible ocular motility disorder [6]. Some authors have reported profuse bleeding when attempting to perform incisional biopsy [3, 7, 9]. The use of systemic steroids to shrink the tumour can be of limited value, producing a poor response due to encapsulation and presence of cavernous elements [1, 7]. Moreover, injecting intralesional steroids might result in a retrobulbar haemorrhage [6]. Observation with conservative management is also an option [9].

The recurrence rates of IMH after incomplete excision are reported to be 20% for the capillary type, 9% for the cavernous type and 28% for the mixed type of IMH [7].

In our case, the superior rectus was involved; histopathological examination revealed the tumour to be of a capillary-type. Due to the unpredictable surgical results and the potential consequence of causing irreversible motility disturbance, total excision was not considered as treatment option at the time of presentation. Furthermore, our case demonstrated a recurrence of the tumour, and exceptionally, an intracranial extension, that has never been reported previously in the literature.

In conclusion, IMH of the extraocular muscles are extremely rare, but it should be considered in the differential diagnosis of an extraocular muscle enlargement. The diagnosis is usually difficult to establish without biopsy. Due to its rarity, the most appropriate treatment for IMHs of the extraocular muscles remains challenging. No extension to surrounding tissues or intracranial extension was acknowledged until now.

## Data Availability

Not applicable.

## Abbreviations

IMH: Intramuscular haemangioma
BCVA: Best corrected visual acuity
IOP: Intraocular pressure
EOM: Extraocular muscle
CT: Computed tomography
MRI: Magnetic resonance imaging
TA: Triamcinolone acetate
VA: Visual acuity
MR: Medial rectus
LR: Lateral rectus
SR: Superior rectus
IR: Inferior rectus
SO: Superior oblique
